# High-quality draft genome sequence of endophytic *Kluyvera ascorbata* strain PCL31 isolated from *Piper crocatum* (red betel)

**DOI:** 10.12688/f1000research.183746.1

**Published:** 2026-07-06

**Authors:** Elizabeth Sidhartha, Rahadian Pratama, Ernst Richard Ng, Irene Nathania Christiawan, Yeshaya Valentine, Rio Hermantara, I Made Artika, Okti Nadia Poetri, Mega Safithri

**Affiliations:** 1 Department of Biochemistry, Graduate School, IPB University, Bogor, West Java, Indonesia; 2 Department of Biomedicine, School of Health and Life Sciences, i3L University, Jakarta, Indonesia; 3 Biomolecule Division, Department of Biochemistry, IPB University, Bogor, West Java, Indonesia; 4 Department of Infectious Diseases and Public Health, School of Veterinary Medicine and Biomedical Sciences, IPB University, Bogor, West Java, Indonesia; 5 Bioanalysis Division, Department of Biochemistry, IPB University, Bogor, West Java, Indonesia; 6 Tropical Biopharmaca Research Center, IPB University, Bogor, West Java, Indonesia

**Keywords:** Kluyvera ascorbata, Piper crocatum, bacterial endophyte, Oxford Nanopore, genome mining, biosynthetic gene clusters, resistance genes

## Abstract

Endophytic bacteria within medicinal plants are a largely untapped reservoir of bioactive compounds and adaptive genetic traits. *Piper crocatum* (red betel), a Southeast Asian medicinal herb rich in flavonoids, alkaloids, and phenolics, offers a chemically selective niche for endophytes, yet the genomes of its microbial associates remain poorly characterised. *Kluyvera ascorbata* occurs as a plant-associated endophyte and an opportunistic human pathogen, and its chromosomal *bla*KLUA gene is the recognised progenitor of the clinically important CTX-M-2 β-lactamase sublineage. We report a high-quality draft genome of endophytic strain PCL31, isolated from surface-sterilised *Piper crocatum* tissues collected in Bogor, Indonesia. Whole-genome sequencing used the Oxford Nanopore PromethION platform, with *de novo* assembly in Flye, polishing in Medaka, and quality assessment by QUAST and BUSCO. The assembly spans 4,978,299 bp across eight contigs (N50 1.45 Mbp; GC content 54.26%; 100% BUSCO completeness, *enterobacterales_odb10*, n = 440). Species identity was confirmed by digital DNA–DNA hybridisation (dDDH 88.6% against the type strain *K. ascorbata* ATCC 33433) and average nucleotide identity (ANI 98.6% against reference genome NZ_CP096201.1). Annotation identified 4,579 protein-coding sequences, 22 ribosomal RNA, 88 transfer RNA, and 81 non-coding RNA genes, and no CRISPR/Cas systems. antiSMASH 8.0 predicted four biosynthetic gene clusters (BGCs): a 53.8 kb non-ribosomal peptide synthetase (NRPS)–metallophore cluster matching the conserved enterobactin locus (MIBiG similarity 0.83), plus three low-similarity clusters (≤ 0.50) of uncertain novelty — a ribosomally synthesised and post-translationally modified peptide (RiPP)-like cluster, a terpene-precursor cluster, and an azole-containing RiPP. CARD-RGI profiling returned one Perfect hit ( *bla*CTX-M-2) and 25 Strict hits, the latter dominated by intrinsic *Enterobacteriaceae* efflux and target-modification genes; phenotypic confirmation was not performed. This genome provides a resource for bioprospecting secondary metabolites from medicinal-plant endophytes and underscores the value of plant-associated isolates for biosafety and antimicrobial-resistance surveillance in opportunistic pathogens.

## Introduction

Endophytes, mainly bacteria and fungi, colonise plant tissues internally throughout part or all of their life cycle without producing obvious disease symptoms in the host plant ( Ali et al., 2024). They represent a largely untapped reservoir of bioactive compounds and adaptive genetic traits ( Ali et al., 2024; Tsipinana et al., 2023). Endophytes play a central role in plant fitness by enhancing nutrient uptake, generating phytohormones, promoting tolerance to environmental challenges, and protecting host plants from pathogens ( Tsipinana et al., 2023). Endophytes are also able to produce bioactives such as antibacterial, anticancer, antioxidant, and anti-inflammatory metabolites that may be of medicinal and commercial interests ( Hashem et al., 2023; Wang et al., 2025). Medicinal plants are considered significant endophyte reservoirs because their diverse secondary metabolites impose continuous chemical selective pressures that may favour particular adaptive features, such as detoxification mechanisms, antimicrobial resistance, stress-responses, and specialised metabolic systems ( Salvi et al., 2022; Singh et al., 2022; Tsipinana et al., 2023).

Red betel ( *Piper crocatum*) is widely used in Indonesian and broader Southeast Asian traditional medicine ( Heliawati et al., 2022). Its leaves are rich in flavonoids, alkaloids, and phenolic compounds that confer numerous biological activities, including antibacterial, antifungal, antioxidant, anti-inflammatory, antidiabetic, and wound-healing properties ( Gong et al., 2021; Heliawati et al., 2022; Purnama et al., 2023; Safithri et al., 2025). This complex phytochemistry suggests a niche under which endophytic bacteria may evolve unique metabolic and defensive capabilities, yet the genomic features of *Piper crocatum* endophytes remain poorly characterised.

Among the *Enterobacteriaceae* recovered from plant tissues, *Kluyvera ascorbata* is of particular interest because of its dual ecological-clinical profile. The species occurs as a plant-associated endophyte but also acts as an opportunistic human pathogen, and its chromosomal *bla*KLUA gene is recognised as the progenitor of the clinically important CTX-M extended-spectrum β-lactamase lineage ( Luong et al., 2025; Rodriguez & Gutkind, 2024; Yu et al., 2023). This dual ecological–clinical identity gives *K. ascorbata* additional significance beyond bioprospecting: environmental and plant-associated bacteria are increasingly recognised as overlooked reservoirs of resistance determinants that may be transferred to clinical pathogens through horizontal gene transfer ( Huang et al., 2024; Michaelis & Grohmann, 2023), making the characterisation of resistance-associated genes in such organisms important for both evolutionary insight and biosafety assessment.

Whole-genome sequencing offers a unified framework to address both dimensions simultaneously, enabling the identification of biosynthetic gene clusters, virulence determinants, and antimicrobial resistance genes that are routinely missed by conventional phenotypic approaches, and supporting prediction of metabolic potential for bioprospecting ( Liu et al., 2022; Meesil et al., 2023; Tamang et al., 2024). Despite the relevance of *K. ascorbata* to both medicinal-plant microbiomes and AMR surveillance, no complete genome characterisation of an endophytic *Kluyvera ascorbata* from *Piper crocatum* has been reported, leaving its biosynthetic capacity, adaptive traits, and resistance repertoire unexplored. Here, we present a high-quality draft genome of an endophytic *Kluyvera ascorbata* isolated from surface-sterilised *Piper crocatum* tissues. The aims are: (i) to deposit a publicly accessible reference genome for an endophytic *Kluyvera ascorbata* from a tropical medicinal plant, (ii) to catalogue its biosynthetic gene cluster inventory, and (iii) to profile its predicted antimicrobial resistance gene complement to inform biosafety and AMR surveillance.

## Methods

### Bacterial isolation and culture

A healthy plant of *Piper crocatum* was collected from Bogor, Indonesia. The plant was identified by Dr. Joeni Setijo Rahajoe (Herbarium Bogoriense, BRIN) by morphological comparison with an authenticated reference specimen of the same species held at the Herbarium Bogoriense (BO), Directorate of Scientific Collection Management, National Research and Innovation Agency (BRIN), Cibinong, Indonesia (specimen accession no. BO-1475717). Plant tissues (leaves, stems, and roots) were thoroughly washed under running tap water, followed by surface sterilisation using 70% ethanol for two minutes, 5% sodium hypochlorite for five minutes, 70% ethanol for 30 seconds, and rinsed four times with sterile distilled water. The sterilised tissues were used for further processing. Tissues were macerated and segmented prior to enrichment in tryptic soy broth (TSB) for four hours in a shaker incubator with 180 RPM at 37 °C. The suspension was then spread onto tryptic soy agar (TSA) supplemented with amphotericin B, and incubated in a 37 °C incubator for 20 hours for bacterial isolation. Distinct bacterial colonies were subcultured onto fresh TSA plates several times until pure isolates were obtained. The isolate used in this study was designated PCL31, preserved at −80 °C in 25% ( *v/v*) glycerol, and deposited in the i3L University microbial culture collection under the strain designation PCL31. The strain is available from the corresponding author (Mega Safithri; safithri@apps.ipb.ac.id) upon reasonable request for non-commercial research use, subject to institutional approval and a completed material transfer agreement. To confirm endophytic origin, 100 μL of the final rinse water was plated on TSA in parallel; no colonies were recovered after 48 h at 37 °C.

### DNA extraction and sequencing

The pure isolates were cultured in tryptic soy broth (TSB) for 24 hours in a shaker incubator at 180 RPM, 37 °C. Then, the pellet of the pure isolate obtained from the cultured broth was centrifuged at 4000 × g for 5 minutes. The pellet was further processed through lysis and purification to isolate genomic DNA using the spin-column method from the gSYNC™ DNA extraction kit (Geneaid Biotech Ltd., Taiwan). Extracted DNA samples were evaluated using gel electrophoresis (1% tris borate-EDTA agarose w/v) for integrity, and NanoDrop Lite Plus microvolume spectrophotometer (Thermo Fisher Scientific, USA) as well as Quantus fluorometer (Promega, USA) equipped with the QuantiFluor ONE dsDNA system, for concentration and purity.

The DNA library was prepared for PromethION ONT using the SQK-NBD114.24 ligation sequencing kit according to the manufacturer’s recommendation (Oxford Nanopore Technology, UK). Kits from NEBNext, FFPE Repair Mix (NEB M6630, UK) and Ultra II End Repair Module (NEB E7546, UK) were employed in the library preparation. In brief, total gDNA was repaired using an end-prep enzyme mix (NEB M6630 & NEB E7546), generating DNA with 5′ phosphorylated, 3′ dA-tailed ends. Barcodes were ligated with an ONT-compatible adapter. The library was quantified with a Quantus Fluorometer (Promega, USA) before loading onto the FLO-PRO114M flow cell. Sequencing control was conducted in MinKNOW v23.04.6 following the default sequencing run from ONT (72 h). The output raw reads were set to POD5 for a separate basecalling process.

### Sequence processing and assembly

The raw POD5 data generated was basecalled using Dorado v0.8.2 super accuracy model and the resulting BAM output was converted to FASTQ using Samtools v1.21 ( Danecek et al., 2021). Next, the raw FASTQ file was assessed using NanoPlot v1.42.0 to analyze long-read ONT whole genome sequences ( De Coster & Rademakers, 2023). A pre-filtering step with Filtlong v0.2.1 ( https://github.com/rrwick/Filtlong) with min-length 1 kb and min-Q 10 did not improve the assembly N50, BUSCO completeness, or estimated coverage in pilot tests, and was therefore omitted from the final pipeline. Reads were then assembled de novo using Flye v2.9.5 ( Kolmogorov et al., 2019) to generate a whole-genome sequencing draft of the isolate in FASTA format. Parameters included *--nano-hq --genome-size 5 m --iterations 3 --threads 4.* The draft assembly was then polished using Medaka consensus v2.0.1 to enhance sequence accuracy and correct residual errors. The quality of the polished assembly, including total length, GC content, and N50, was evaluated using the quality assessment tool for genome assembly (QUAST) v5.3.0 ( Gurevich et al., 2013). Genome completeness of the polished assembly was assessed using the benchmarking universal single-copy orthologs (BUSCO) v5.8.0 ( Manni et al., 2021) against the *enterobacterales_odb10* lineage dataset (n = 440 single-copy orthologs), where assemblies with >95% completeness and < 5% contamination were considered high-quality.

### Contig topology and replicon characterisation

Assembly graph topology was inspected from the Flye *assembly_info.txt* output. Plasmid replicons were screened with PlasmidFinder v2.1 against the *Enterobacterales* database (≥95% identity, ≥60% coverage; Carattoli et al., 2014), and molecule classification (chromosome vs. plasmid) and mobility typing were performed with MOB-suite v3.1.9 using mob_recon defaults ( Robertson & Nash, 2018). Per-contig statistics (length, GC content, coverage, circularity, and replicon/molecule assignment) are provided in Underlying Data ( Sidhartha et al., 2026).

### Taxonomic and phylogenomic analysis

Whole genome-based taxonomic analysis was carried out on the type (strain) genome server (TYGS) ( Meier-Kolthoff & Göker, 2019) by submitting the polished genome assembly and utilizing an unrestricted query. The TYGS webserver performed pairwise comparisons of genome sequences against the TYGS database to yield species identification, DNA-DNA hybridization (dDDH) score, and phylogenetic inference. A genome BLAST distance phylogeny (GBDP) tree was also generated.

The result was further confirmed using FastANI v1.34 ( Jain et al., 2018) against the reference *Kluyvera ascorbata* complete-genome assembly NZ_CP096201.1 (strain SK) retrieved from NCBI RefSeq; the species type strain is ATCC 33433 (assembly JMPL00000000), used for the TYGS dDDH comparison above. The average nucleotide identity (ANI) was calculated by the mean percentage of identical nucleotides across all shared regions between the obtained and reference sequences. Results were interpreted based on the established ANI threshold (>90%).

### Functional annotation and genome mining

Genome annotation was performed using Bakta v1.9.4 ( Schwengers et al., 2021) utilizing Bakta database v5.0_2023-03-20 and AMRFinderPlus database v3.12–2024-05-02.2.

BGCs were identified using antiSMASH 8.0 ( Blin et al., 2025) in relaxed detection mode with KnownClusterBlast, ClusterBlast, SubClusterBlast, MIBiG comparison, and ClusterCompare modules enabled; references were drawn from MIBiG v4.0. Similarity scores represent the aggregated region-level score from antiSMASH (range 0–1), where higher values reflect greater gene content and synteny conservation with the reference cluster; scores ≥0.80 typically indicate clusters encoding homologues of the reference compound, whereas scores ≤0.50 were interpreted as putative, low-confidence matches.

Antimicrobial resistance gene (ARG) profiling was done using the Comprehensive Antibiotic Resistance Database (CARD) v4.0.1 Resistance Gene Identifier (RGI) v6.0.5( Alcock et al., 2023), selecting for Perfect and Strict hits, and specifying the input as high quality/coverage assembly.

The assembled genome was screened for CRISPR arrays and Cas operons using the CRISPRCasFinder ( Couvin et al., 2018) utilizing the default settings.

### Genome map construction

Genome map was visualized using Proksee ( Grant et al., 2023) to generate a circular genome map depicting sequence features, contig boundaries, and genomic architecture. BGCs and ARGs coordinates from their respective tools were manually converted into a location map as.bed file and included as separate forward- and reverse-strand tracks.

## Results

The ONT sequencing generated 117,809 reads totalling 174.6 Mbp, with a read N50 of 3,007 bp and 75% of reads >Q10 ( Table 1). Based on an estimated genome size of ~5.0 Mb for *Kluyvera ascorbata*, this corresponds to an estimated average depth of coverage of approximately 35×. The polished assembly comprised 4.98 Mbp across eight contigs (assembly N50 = 1.45 Mbp; Table 1). All eight contigs were classified as chromosomal by MOB-suite, and PlasmidFinder detected no plasmid replicons (≥95% identity, ≥60% coverage); no plasmids were therefore identified in strain PCL31 (Underlying Data). The fragmentation into eight chromosomal contigs likely reflects unbridged rRNA operons: the genome encodes 22 rRNA genes, corresponding to seven rRNA operons (21 genes) plus one additional 5S rRNA (Bakta annotation, Table 2), with each operon spanning ~5–6 kb that a read N50 of 3 kb cannot span unambiguously. Despite this fragmentation, BUSCO recovered 100.0% of single-copy orthologs from the *enterobacterales_odb10* lineage with 0% duplication and 0% fragmentation, indicating that the chromosomal gene space is fully represented.

**Table 1.** Genome assembly quality assessment.

Parameter	Assembled genome
Genome size (bp)	4,978,299
Number of contigs	8
Number of plasmids	0
Assembly N50 (bp)	1,450,225
GC content (%)	54.26
Genome completeness (%)	100.0
Duplication (%)	0.0
Fragmentation (%)	0.0
Largest contig (bp)	1,748,720
Estimated sequencing depth (X)	35

**Table 2.** General genome features and annotation summary.

Gene type	Number of genes
Protein coding genes (CDS)	4,579
rRNA genes	22
tRNA genes	88
tmRNA	1
ncRNA genes	81
Biosynthetic gene clusters (antiSMASH 8.0)	4
Antimicrobial resistance genes (CARD-RGI; Perfect + Strict)	26 (1 Perfect, 25 Strict)
CRISPR/Cas	0

### Phylogenetic analysis

TYGS species identification using the whole genome assembly suggested *Kluyvera ascorbata*, as indicated by the dDDH value of 88.6% with the reference strain *Kluyvera ascorbata* ATCC 33433 ( Figure 1). The identity was confirmed with the ANI score of 98.6% between the sample and *Kluyvera ascorbata* reference sequence (NCBI RefSeq NZ_CP096201.1). The next-closest TYGS match was *Kluyvera cryocrescens* (dDDH = 54.3%), well below the 70% species-delineation threshold, confirming species-level assignment to *Kluyvera ascorbata.*

**Figure 1.**
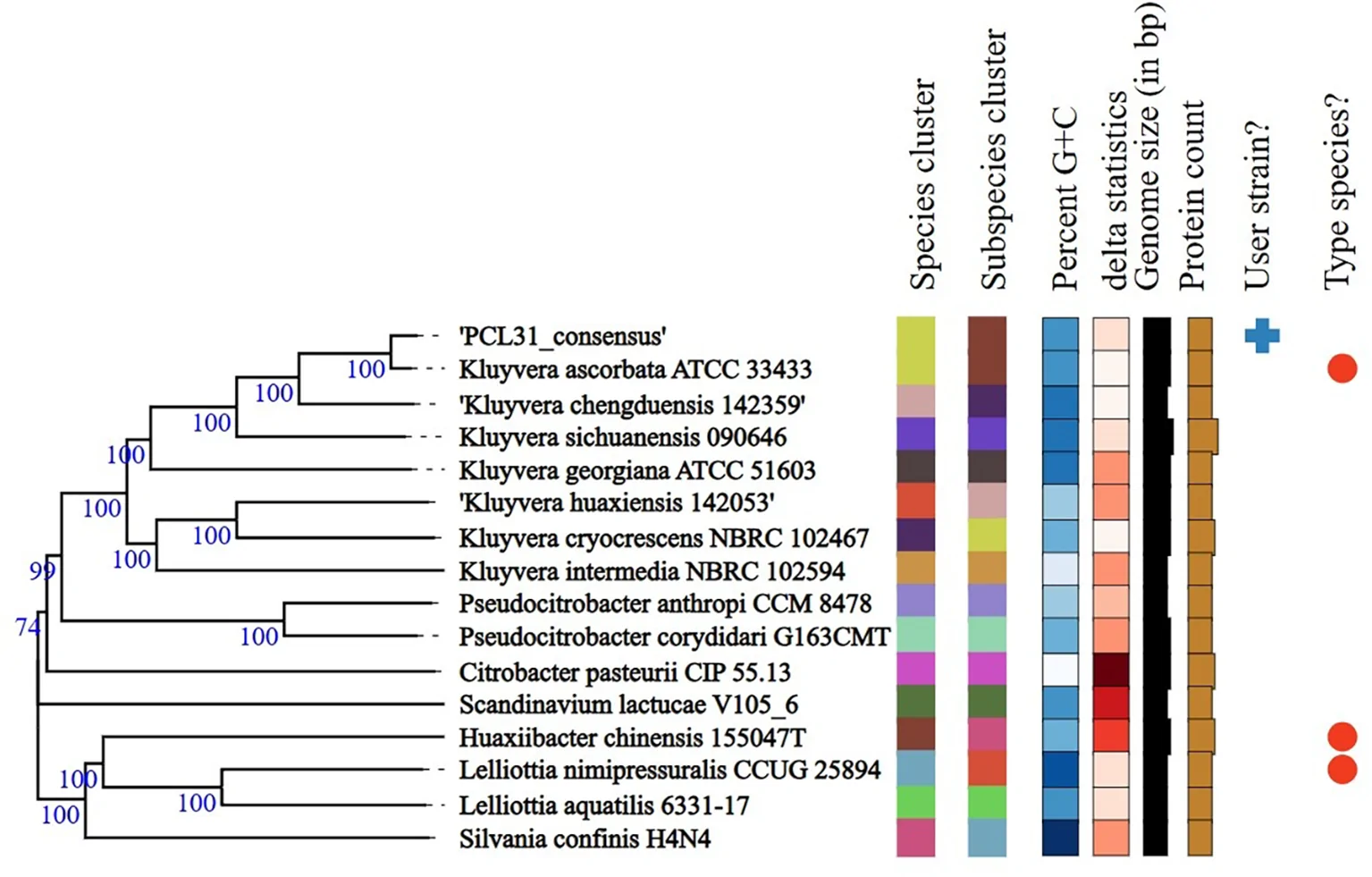
Genome BLAST distance phylogeny (GBDP) tree generated by TYGS for *Kluyvera ascorbata* strain PCL31 versus the closest type strains. Branch lengths are scaled in GBDP distance formula. Numbers at each branch are GBDP pseudo-bootstrap support values from 100 replications.

### Biosynthetic gene clusters

Genome mining with antiSMASH predicted four putative biosynthetic gene clusters (BGCs) in the assembly ( Table 3). The 53.8 kb NRP-metallophore/NRPS cluster on contig_1 corresponded to the canonical enterobactin biosynthesis locus of *Escherichia coli* K-12 (MIBiG BGC0002476; similarity = 0.83), consistent with the presence of this deeply conserved siderophore pathway across *Enterobacteriaceae.* The remaining three clusters — a RiPP-like, a terpene-precursor, and an azole-containing RiPP region — returned only low-similarity matches (scores ≤0.50) to MIBiG references from phylogenetically distant taxa, and therefore represent clusters with no close match in MIBiG. Low similarity may reflect either true biosynthetic novelty or under-representation of *Kluyvera*-associated clusters in current reference databases, requiring further metabolomic validation.

**Table 3.** Biosynthetic gene clusters (BGCs) predicted in the *Kluyvera ascorbata* genome assembly and their closest matches in the MIBiG 4.0 database. Abbreviations: NRP, non-ribosomal peptide; NRPS, non-ribosomal peptide synthetase; RiPP, ribosomally synthesised and post-translationally modified peptide.

Region	Location (contig: start-end)	Size (kb)	Cluster type	Closest MIBiG hit	Reference compound (organism)	Similarity score
1	contig_3: 1,175,821–1,186,444	10.6	RiPP-like	BGC0002338	sorangipyranone ( *Myxococcales* bacterium)	0.20
2	contig_2: 175,918–196,817	20.9	Terpene-precursor	BGC0001361	sodorifen ( *Serratia plymuthica*)	0.50
3	contig_1: 1,412,274–1,438,581	26.3	Azole-containing RiPP	BGC0001285	pseudopyronine A/B ( *Pseudomonas putida*)	0.36
4	contig_1: 1,693,050–1,746,840	53.8	NRP-metallophore/NRPS	BGC0002476	enterobactin ( *Escherichia coli* K-12)	0.83

### Antimicrobial resistance genes

CARD RGI screening of the *Kluyvera ascorbata* assembly returned 26 AMR determinants (1 Perfect, 25 Strict). The sole Perfect hit was *bla* CTX-M-2, located on the chromosomal contig_4 and with a gene length of 873 bp, which is consistent with *Kluyvera ascorbata* being the recognised chromosomal progenitor of the CTX-M-2 β-lactamase lineage. Strict hits were dominated by efflux-pump components, including ten resistance-nodulation-cell division (RND)-family genes ( *acrA*, *acrB*, *acrD*, *adeF*, *baeR*, *CRP*, *marA*, *mdtB*, *mdtC*, *rsmA*) and a MarR-overexpression variant of the AcrAB–TolC system, three major facilitator superfamily (MFS) pumps ( *emrR*, *KpnH*, *leuO*), two small multidrug resistance (SMR) pumps ( *KpnE*, *KpnF*), and one ATP-binding cassette (ABC) pump ( *msbA*). Target-alteration determinants comprised *vanL*, *ArnT*, *PmrF*, two paralogues of EF-Tu carrying R234F, *UhpT* (E350Q), and *PBP3* (D350N/S357N), and an additional inactivation enzyme, *FosA3*, was also recovered. Collectively, the predicted resistome spans β-lactams, fluoroquinolones, aminoglycosides, macrolides, tetracyclines, glycopeptides, fosfomycin, polymyxins, aminocoumarins, phenicols, rifamycins, and disinfectants. The Strict-hit complement comprises predominantly intrinsic *Enterobacteriaceae* efflux systems and chromosomal target-modification genes whose contribution to clinical resistance phenotype cannot be inferred from sequence alone. Phenotypic resistance testing (MIC) was outside the scope of this Genome Note and is identified here as a limitation.

No CRISPR/Cas component was detected using the CRISPR/Cas Finder tool. The schematic of a circular map of the genome, including the annotated BGCs and ARGs, is presented in Figure 2.

**Figure 2.**
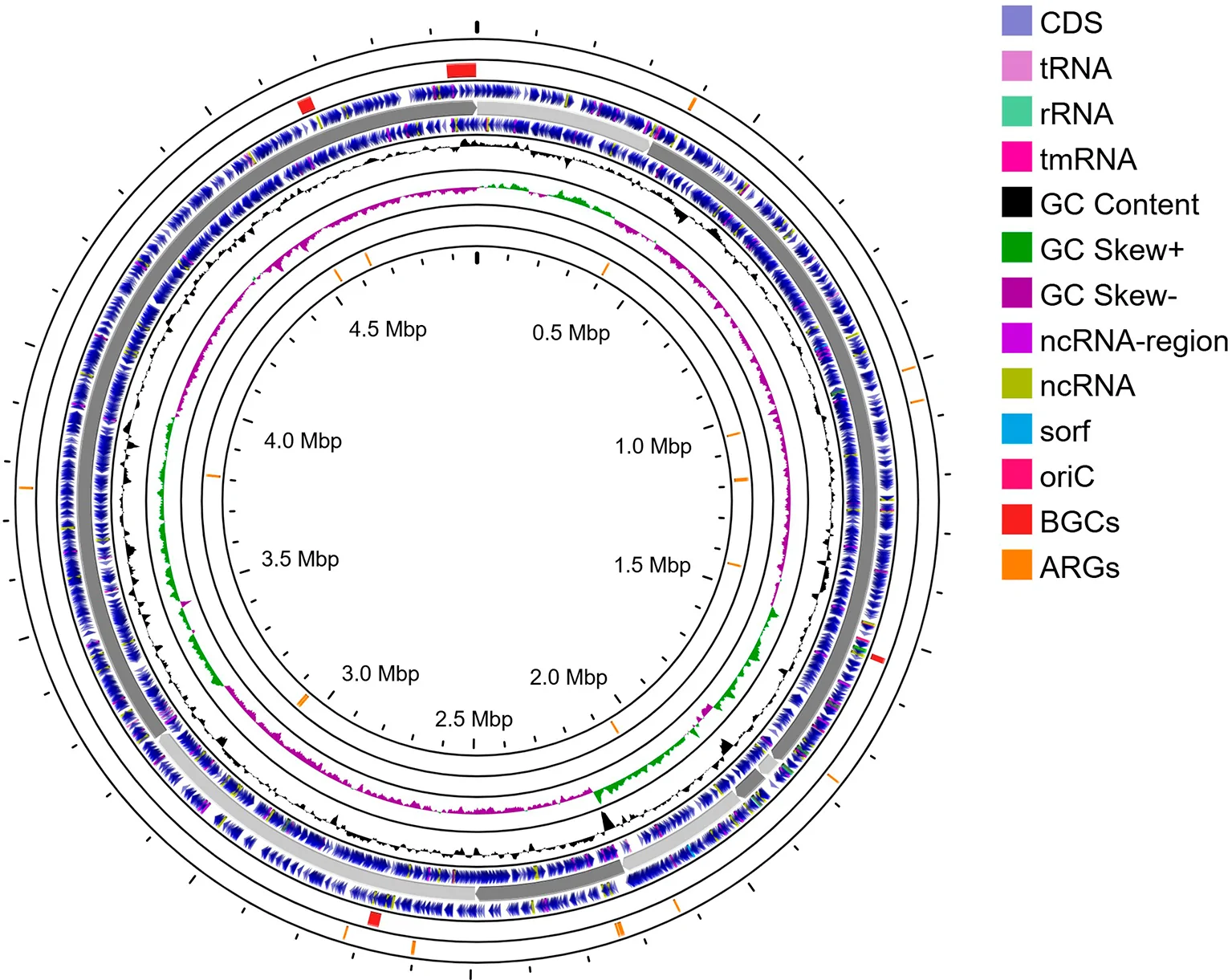
A circular map of the endophytic *Kluyvera ascorbata* genome. Tracks from the outside to the inside: ARGs on the forward strand; BGCs on the forward strand; CDS on the forward strand; contigs; CDS on the reverse strand; GC content; BGCs on the reverse strand (none detected); and ARGs on the reverse strand. Abbreviations: ARGs, antimicrobial resistance genes; BGCs, biosynthetic gene clusters; CDS, coding sequences.

In summary, this Genome Note describes a high-quality draft genome of an endophytic *Kluyvera ascorbata* strain PCL31 isolated from the medicinal plant *Piper crocatum*, together with its annotation, predicted biosynthetic gene clusters, and predicted antimicrobial resistance gene complement. The genome is made publicly available as a resource to support future functional, metabolomic, and antimicrobial resistance studies; detailed analysis and interpretation of these features are beyond the scope of this Genome Note.

## Ethical considerations

The article does not concern animals or human subjects nor carries ethical issues.

## Data Availability

The reference specimen used for taxonomic identification of the plant is held at the Herbarium Bogoriense (BO), Directorate of Scientific Collection Management, National Research and Innovation Agency (BRIN), Soekarno Science and Technology Area, Jl. Raya Jakarta–Bogor Km. 46, Cibinong, Bogor 16911, Indonesia, under accession number BO-1475717 ( Herbarium Bogoriense, n.d). The specimen can be accessed by contacting the herbarium. Raw Oxford Nanopore reads have been deposited in the NCBI Sequence Read Archive under accession SRR38898151 (BioProject PRJNA1472441, BioSample SAMN60497367) ( NCBI, 2026). on Figshare: Underlying data for “High-quality draft genome sequence of endophytic *Kluyvera ascorbata* strain PCL31 isolated from *Piper crocatum* (red betel)” https://doi.org/10.6084/m9.figshare.32499168 ( Sidhartha et al., 2026). This project contains the following underlying data:
• Contig statistics • Bakta annotation files (.gff3, .gbff, .faa, .fna). • QUAST report. • BUSCO full table. • TYGS report. • FastANI output. • antiSMASH 8.0 region-level output (.gbk and HTML report). • CARD-RGI output (.json and.txt). • MOB-Recon output. • PlasmidFinder output. Data are available under the terms of the Creative Commons Attribution 4.0 International license (CC BY 4.0).
