# Supplementary material for: High-Quality Draft Genome Sequence of Endophytic Kluyvera Ascorbata Strain PCL31 Isolated from Piper Crocatum (red betel)
Source: F1000Res. 2026 Aug 21;15:1073. Originally published 2026 Jul 6. [Version 2] doi: 10.12688/f1000research.183746.2 (PMC13687735; doi:10.12688/f1000research.183746.2)
Supplement: Peer review history

# Reviewer report: Reviewer response for version 2

**DOI:** 10.5256/f1000research.208553.r527708

**Published:** 2026-10-27

**Authors:** Hernan A Lorenzi

**Affiliations:** 1 National Institutes of Health, Bethesda, Maryland, USA

In their manuscript entitled “High-Quality Draft Genome Sequence of Endophytic *Kluyvera ascorbata* Strain PCL31 Isolated from *Piper crocatum* (red betel),” Sidhartha et al. describe the genome sequencing, assembly, annotation, and partial characterization of an endophytic bacterium isolated from *Piper crocatum*. The manuscript is well written, the methods are appropriate, and the sequencing data, genome assembly, and annotations have been made publicly available. The findings will be of interest to researchers studying plant-associated bacteria and microbial genomics. The authors have appropriately addressed all reviewers’ concerns, and I therefore approve this revised version.

 Minor editorial suggestion:

 Please clarify the criteria used to designate the assembly as a “high-quality draft” and cite the relevant genome quality standards. If this designation cannot be adequately supported, consider using “draft genome” throughout the manuscript, including the title.

Are the datasets clearly presented in a usable and accessible format, and the assembly and annotation available in an appropriate subject-specific repository?

Yes

Are sufficient details of the sequencing and extraction, software used, and materials provided to allow replication by others?

Yes

Are the rationale for sequencing the genome and the species significance clearly described?

Yes

Are the protocols appropriate and is the work technically sound?

Yes

Reviewer Expertise:

Parasite genomics, Bioinformatics, Microbiome

I confirm that I have read this submission and believe that I have an appropriate level of expertise to confirm that it is of an acceptable scientific standard.

# Reviewer report: Reviewer response for version 2

**DOI:** 10.5256/f1000research.208553.r527702

**Published:** 2026-10-21

**Authors:** Francisco Callejas-Hernández

**Affiliations:** 1 Johns Hopkins University Bloomberg School of Public Health, Baltimore, Maryland, USA

The manuscript by Sidharta et al. describes a new genome assembly of the endophytic Kluyvera ascorbata (strain PCL311) isolated from Piper Crocatum. With approximately 35X coverage of ONT reads, the authors obtained an assembly of eight contigs totaling 4.9 Mb, with a G+C content of 54%.26. The authors also included genome annotation and completeness estimates that, overall, confirm the assembly is of acceptable quality.

 I find the manuscript's main finding interesting for indexing; however, although other reviewers have addressed some weaknesses, I see a few remaining issues that must be considered before indexing.

**Major comments:**

 Today, given the availability of long-read, high-quality sequencing technologies, I consider an assembly of eight contigs for a 5 Mb genome acceptable, not high quality. So, I suggest removing this overstatement from the title and including the average read quality in the text.

 Many genome sequences for Kluyvera ascorbata are already available, some at chromosome-level. Are they syntenic? If so, I suggest using them to scaffold the new genome and provide more accurate estimates of sequence completeness (including non-coding regions).

 Second, the introduction section acknowledges the many genome sequences for this bacterium already available in public databases and explains why this new sequence might be relevant. However, I still do not see the results supporting this argument. I suggest providing more evidence for why a new genome was needed, e.g., SNPs relative to other strains, gene loss/gain, etc. How divergent is this new genome compared to others? Is that what the authors expected?

 I agree with the authors that longer reads could produce a less fragmented genome. Could you hypothesize why such a short read length was obtained? Is this a DNA extraction problem?

 I consider that Figure 1 needs reformatting for better interpretability, along with an expanded figure legend. I don’t think the two columns for species and subspecies clusters are useful or necessary. The color code for “Percent G+C” is missing; what does the white color mean? high? low? Same for “User strain?” and “Type species?”. I suggest removing these last two.  

 Same issue with Figure 2. It has too much unnecessary information, which makes it difficult to interpret. I.e the two CDS circles can be replaced by gene density. What do the light and dark gray circles indicate? Is GC skew relevant at all? I suggest removing it. The others are difficult to see (ncRNA, sorf, oriC, etc). The figure legend must be improved.

 Minor comments:

 None.

Are the datasets clearly presented in a usable and accessible format, and the assembly and annotation available in an appropriate subject-specific repository?

Yes

Are sufficient details of the sequencing and extraction, software used, and materials provided to allow replication by others?

Yes

Are the rationale for sequencing the genome and the species significance clearly described?

Partly

Are the protocols appropriate and is the work technically sound?

Yes

Reviewer Expertise:

Bioinformatics, genomics, transcriptomics, parasitology

I confirm that I have read this submission and believe that I have an appropriate level of expertise to confirm that it is of an acceptable scientific standard, however I have significant reservations, as outlined above.

# Reviewer report: Reviewer response for version 2

**DOI:** 10.5256/f1000research.208553.r523808

**Published:** 2026-10-08

**Authors:** Arun Prasanna Nagarajan

**Affiliations:** 1 King Abdullah University of Science and Technology (KAUST), Thuwal, Saudi Arabia

**General Evaluation**

 This is a well-conducted and valuable genome note reporting the high-quality draft genome of an endophytic *Kluyvera ascorbata* strain (PCL31) isolated from the medicinal plant *Piper crocatum*. The authors have done a commendable job responding to many of the initial concerns raised by Reviewer 1 (Dr. Mushtak T. S. Al-Ouqaili), particularly regarding contamination assessment (implementing CheckM2), clarifying the ANI species boundaries, refining the discussion around the *bla*KLUA gene in progenitor context (CTX-M-2), and toning down unsupported claims of biosynthetic novelty. It is also particularly appreciated to have transparently included about the “Use of Generative AI” section.

 However, a few critical points and nuances remain unaddressed or require further refinement to satisfy rigorous standards for indexing. Below is constructive feedback addressing few gaps.

**Specific Points to Address**

**1. Introduction: Few ambiguities**

- *“Endophytes, mainly… host plant (Ali et al., 2024)”*.  The opening phrasing ("without producing obvious disease symptoms)” correctly notes that endophytes do not cause active disease. However, to ensure clarity and avoid any unintended inference of latent pathogenicity, it would be helpful to explicitly frame endophytes from the outset as mutualistic, commensal, or symbiotic partners (such as Plant Growth-Promoting Bacteria/PGPR) rather than solely as non-symptomatic entities, better aligning with their established roles in plant fitness.
- Para 3 – *“Among the Enterobacteriaceae recovered from plant tissues”, *The transition to discussing *Enterobacteriaceae* and *Kluyvera ascorbata* feels abrupt and lacks a clear contextual bridge from the preceding discussion on *Piper crocatum* phytochemistry. Furthermore, since this study is based on the targeted culture-dependent isolation of a single strain (`PCL31`) and subsequent whole-genome sequencing rather than a metagenomic survey, the text should clarify this focus. Please smooth out this transition to explicitly link why an *Enterobacteriaceae* endophyte is being investigated in the context of this specific medicinal plant.

**2. Methodological Precision: Library Preparation Kits & Basecalling Parameters**

- **The Issue:** While the authors state that the ONT library was prepared using the SQK-NBD114.24 kit alongside NEB modules (FFPE Repair Mix and Ultra II End Repair Module), they do not specify the exact input amounts or molar ratios used for barcoding ligation, nor do they clarify the exact basecalling configuration parameters beyond naming the Dorado super-accuracy model and version (v0.8.2).

- **Recommendation:** Provide the exact DNA mass input used into the end-prep reaction (e.g., how many nanograms from the 400 ng total minimum was actually committed to the reaction) and specify whether the Dorado model was run with CPU or GPU acceleration, as well as the specific model file/tag used, to ensure full protocol replicability. What was the cut-off you used to define high-molecular-weight DNA? What was the size used as the input? Finally, what was the average read length output achieved?

**3. Amplicon / Molecular Verification of Surface Sterilization Controls**

- **The Issue:** Reviewer 1 noted that plating the final rinse water is insufficient to entirely rule out persistent epiphytes or internal contamination compared to tissue imprinting or PCR-based validation. Although the authors responded that the tissue was fully consumed during processing and cannot be tested retrospectively, this limitation is omitted from the main text's discussion of methods.

- **Recommendation:** Explicitly add a brief statement in the *Methods* or *Discussion/Limitations* section acknowledging that while rinse-water plating showed no growth, culture-independent molecular verification (such as 16S rRNA PCR of pre-maceration tissue imprints) was not performed, which represents a standard limitation for establishing absolute endophytic localization.

**4. Over-Reliance on Short-Read Polishing / Consensus Tools for ONT-Only Data**

- **The Issue:** The authors mention that only a single round of Medaka polishing was performed because Dorado super-accuracy basecalling obviates the need for tools like Racon. While super-accuracy models (such as sup) perform exceptionally well, Oxford Nanopore data—especially at a modest ~35X coverage depth—can still harbour systematic homopolymer length errors or consensus inaccuracies that a single iteration of Medaka may not completely resolve.

- **Recommendation:** Discuss whether a second round of Medaka was tested for convergence (e.g., checking if variant sites plateaued), or explicitly state the rationale for restricting polishing to exactly one round based on coverage limitations.

**5. Biosynthetic Gene Cluster (BGC) Annotation and Contig Boundary Artifacts**

- **The Issue:** The antiSMASH results indicate four BGCs, with three showing very low similarity scores ( *≤0.50*). Although the authors moderated their claims in Version 2 regarding "novelty", they attribute low similarity entirely to database gaps or hypothetical novelty without explicitly checking whether any of these low-scoring clusters are artificially truncated by contig boundaries (i.e., hitting the edge of one of the 8 contigs).

- **Recommendation:** Confirm via the antiSMASH region output whether any of the three low-similarity BGCs (RiPP-like, terpene-precursor, azole-containing RiPP) span across a contig break or sit near a contig edge. If a cluster is truncated at a contig boundary, state this explicitly as a primary technical driver for low similarity scores rather than leaving it to biological speculation.

**6. Resistome Nomenclature and Intrinsic vs. Acquired Annotation Clarity**

- **The Issue:** The CARD-RGI screening returned 26 determinants (1 Perfect, 25 Strict). While the text now rightly calls out that most strict hits are intrinsic efflux or regulatory components, the distinction between *true resistance mutations* (such as specific point mutations in target genes like *PBP3* or *EFS-Tu*) and *efflux overexpression regulators* (like MarR or BaeR variants) requires clearer clinical framing. For instance, specific amino acid substitutions (e.g., D350N/S357N in PBP3) should be explicitly cross-referenced against known functional resistance markers in Enterobacteriaceae to clarify whether they confer baseline insensitivity or act as mere homologs.

- **Recommendation:** Expand the *Antimicrobial resistance genes* results subsection slightly to clarify the exact nature of the target-alteration mutations (e.g., whether the identified mutations are experimentally validated markers of resistance or conserved housekeeping variations typical of the genus *Kluyvera*).

**Summary Recommendation**

 The manuscript is scientifically sound, methodologically rigorous following the Version 2 revisions, and fits the scope of a high-quality Genome Note. Incorporating the minor clarifications above regarding surface sterilization limitations, contig boundary checks for BGCs, and precise resistome mutation context will make the manuscript fully tight for final approval.

Are the datasets clearly presented in a usable and accessible format, and the assembly and annotation available in an appropriate subject-specific repository?

Yes

Are sufficient details of the sequencing and extraction, software used, and materials provided to allow replication by others?

Yes

Are the rationale for sequencing the genome and the species significance clearly described?

Yes

Are the protocols appropriate and is the work technically sound?

Yes

Reviewer Expertise:

Bioinformatics, NGS technologies, Phylogenomics, Molecular Biology, Genome Assembly, Transcriptomics

I confirm that I have read this submission and believe that I have an appropriate level of expertise to confirm that it is of an acceptable scientific standard.

# Reviewer report: Reviewer response for version 1

**DOI:** 10.5256/f1000research.202819.r503971

**Published:** 2026-08-03

**Authors:** Mushtak T. S. Al-Ouqaili

**Affiliations:** 1 Department of Microbiology, University of Anbar, College of Medicine, Ramadi City, Anbar Governorate, Iraq

*Dear Editor,*

*This manuscript introduces a unique concept in an area that is both intriguing and important for medical research. The topic is timely and is likely to engage the journal's readers. Additionally, the manuscript is well-written, presenting ideas clearly and logically.*

*Dear Authors,*

*You are doing excellent work, and this manuscript reflects a great deal of effort and thoughtful research. The study is promising and contributes meaningfully to the field. However, there are a few important points that require further clarification and revision to enhance the overall quality and impact of the manuscript.*

*It is preferred to use the following reference [Ref 1] to support your updated citations.*

*The discussion of Piper crocatum emphasizes its medicinal value but does not sufficiently explain how its phytochemical composition may shape endophytic genome evolution. Expanding this ecological rationale would strengthen the justification for whole-genome sequencing.*

*It is preferred to use the following reference [Ref 2] to support your updated citations.*

*The manuscript links plant-associated bacteria with antimicrobial resistance dissemination through horizontal gene transfer. However, this argument would benefit from additional explanation regarding:*

- *ecological pathways connecting plant microbiomes and clinical settings, *
- *evidence supporting gene exchange, *
- *relevance of Kluyvera as an environmental reservoir of clinically important resistance genes. *

*Otherwise, the transition appears somewhat abrupt.*

*The introduction states that "no complete genome characterisation of an endophytic Kluyvera ascorbata from Piper crocatum has been reported", but this claim should be supported by a more comprehensive literature context. The authors should clearly distinguish their work from previous genome reports of K. ascorbata isolated from environmental, plant-associated, or clinical sources, explaining precisely what knowledge gap this study fills.*

*It is preferred to use the following reference [Ref 3] to support your updated citations.*

*The manuscript should explain why this genome will be valuable beyond the current study—for example, comparative genomics, evolutionary studies, genome mining, natural product discovery, or future surveillance of environmental antimicrobial resistance.*

*The manuscript does not explain why enrichment and bacterial isolation were performed at 37°C, which is considerably higher than the natural environmental temperature of Piper crocatum. This incubation temperature may preferentially recover mesophilic bacteria with potential clinical relevance while excluding other endophytic taxa. The authors should justify this choice or discuss its implications for isolate recovery.*

*Although plating of the final rinse water is reported, the methodology would be stronger if additional evidence of successful surface sterilization were included (e.g., imprint method or molecular verification). The authors should discuss whether plating alone is sufficient to exclude epiphytic contamination.*

*The authors describe DNA integrity assessment but do not report the acceptance criteria used before sequencing.*

*Please specify:*

- *minimum DNA concentration, *
- *A260/A280 ratio, *
- *A260/A230 ratio, *
- *DNA quantity loaded for library preparation. *

*These parameters improve reproducibility.*

*The sequencing workflow is comprehensive, but no information is provided regarding:*

- *total number of reads, *
- *total bases generated, *
- *mean read length, *
- *N50 read length, *
- *sequencing depth (coverage). *

*These values are essential because they directly influence assembly quality.*

*The manuscript states:*

*"assemblies with >95% completeness and <5% contamination were considered high-quality."*

*However, BUSCO evaluates completeness and duplication—not contamination.*

*Contamination is usually estimated using tools such as:*

- *CheckM, *
- *CheckM2, *
- *GUNC, *
- *BlobToolKit. *

*The authors should either:*

- *replace "contamination" with "duplication," or *
- *include an independent contamination assessment. *

*This is one of the most important methodological issues.*

*The manuscript states:*

*"Results were interpreted based on the established ANI threshold (>90%)."*

*This threshold is incorrect for bacterial species delineation.*

*Current consensus is:*

- *95–96% ANI for species boundaries. *

*The manuscript should revise this statement and cite current ANI standards.*

*Only Medaka polishing was performed.*

*Please clarify:*

- *whether multiple polishing rounds were conducted, *
- *whether polishing converged, *
- *why no additional polishing (e.g., Racon) was performed. *

*Although the Bakta version is reported, the manuscript should indicate:*

*annotation mode,*

*genetic code used,*

*whether pseudogenes were predicted,*

*whether default parameters were employed.*

*The reported sequencing depth (~35×) is relatively modest for generating a high-quality Oxford Nanopore-only bacterial genome assembly. Although the BUSCO completeness is excellent (100%), the authors should discuss whether this sequencing depth may have contributed to the fragmented assembly (eight contigs) and whether additional sequencing could improve contiguity.*

*The authors attribute fragmentation entirely to rRNA operons because the read N50 (~3 kb) is shorter than the operon length.*

*However, this explanation remains speculative.*

*Other factors may also contribute, including.*

- *repetitive insertion sequences, *
- *prophages, *
- *tandem repeats, *
- *GC-rich regions. *

*The authors should either provide assembly graph evidence supporting this explanation or moderate the wording.*

*The manuscript reports assembly statistics but provides little context.*

*It would strengthen the Results if the assembly quality (genome size, GC content, contig number, N50) were briefly compared with published Kluyvera ascorbata genomes to demonstrate consistency.*

*The statement*

*"Low similarity may reflect either true biosynthetic novelty..."*

*is speculative.*

*Low similarity in antiSMASH can also result from*

- *fragmented clusters, *
- *incomplete assemblies, *
- *annotation differences, *
- *limited database representation. *

*The manuscript should avoid implying novelty without experimental validation.*

*The manuscript identifies 26 AMR determinants, yet most are regulatory or efflux-associated genes.*

*The wording could lead readers to interpret these as confirmed resistance genes.*

*The authors should emphasize that many identified loci are*

- *intrinsic housekeeping genes, *
- *regulatory genes, *
- *putative contributors to resistance, *

*rather than acquired resistance determinants.*

*The manuscript states*

*"Perfect hit was blaCTX-M-2..."*

*Since Kluyvera species possess chromosomal blaKLU genes that are ancestral to CTX-M enzymes, the authors should clarify whether CARD annotated this locus directly as CTX-M-2, or whether it represents a homologous ancestral chromosomal β-lactamase.*

*This distinction has evolutionary importance.*

*Phenotypic antimicrobial susceptibility testing is mentioned only near the end.*

*Since the Results emphasize antimicrobial resistance genes, the absence of MIC or susceptibility testing should be acknowledged immediately after presenting the CARD results.*

*The absence of CRISPR/Cas systems is reported without interpretation.*

*A brief statement regarding whether CRISPR absence is common in Kluyvera species would improve biological context.*

*The final paragraph appropriately states that detailed interpretation is beyond the scope of the Genome Note.*

*However, several earlier paragraphs already provide biological interpretation (e.g., novelty of BGCs, AMR implications).*

*The manuscript should maintain a consistent balance between descriptive reporting and interpretation.*

*The conclusion should be objective, with further perspective, or should add at least a few sentences about future study/future perspective*

Are the datasets clearly presented in a usable and accessible format, and the assembly and annotation available in an appropriate subject-specific repository?

Yes

Are sufficient details of the sequencing and extraction, software used, and materials provided to allow replication by others?

Yes

Are the rationale for sequencing the genome and the species significance clearly described?

Yes

Are the protocols appropriate and is the work technically sound?

Partly

Reviewer Expertise:

Microbiology, Molecular Biology. Molecular Microbiology. Molecular diagnosis of infectious disease

I confirm that I have read this submission and believe that I have an appropriate level of expertise to confirm that it is of an acceptable scientific standard, however I have significant reservations, as outlined above.

## References

[1] : Recent advances of plant-mediated metal nanoparticles: Synthesis, properties, and emerging applications for wastewater treatment. *Journal of Environmental Chemical Engineering* .2024;12(2) : 10.1016/j.jece.2024.112345 10.1016/j.jece.2024.112345

[2] : Molecular and bacteriological characterization of colistin and carbapenem-resistant nosocomial isolates of Acinetobacter baumannii isolated from different Iraqi hospitals. *Journal of Infection and Public Health* .2026;19(3) : 10.1016/j.jiph.2025.103118 10.1016/j.jiph.2025.103118 41477986

[3] : Molecular characterization and genome sequencing of selected highly resistant clinical isolates of Pseudomonas aeruginosa and its association with the clustered regularly interspaced palindromic repeat/Cas system. *Heliyon* .2025;11(1) : 10.1016/j.heliyon.2025.e41670 10.1016/j.heliyon.2025.e41670 PMC1176134139866497

# Response

**Published:** 2026-08-13

**Authors:** Mega Safithri

**Affiliations:** Department of Biochemistry, IPB University, Bogor, Indonesia

We thank the reviewer for the careful and constructive assessment, and for the encouraging appraisal of the work. The comments have materially improved the manuscript. We have revised the text accordingly and respond point by point below; all changes are incorporated in Version 2.

  

1. Reviewer comment: *The discussion of Piper crocatum emphasizes its medicinal value but does not sufficiently explain how its phytochemical composition may shape endophytic genome evolution. Expanding this ecological rationale would strengthen the justification for whole-genome sequencing.* ​​​​​​​ **Response:** We thank the reviewer for this helpful suggestion. We have expanded the Introduction to make this rationale explicit, noting that the rich phytochemistry of *Piper crocatum* imposes a selective niche that may favour detoxification, efflux, and specialised metabolic traits in resident endophytes, thus providing a concrete justification for whole-genome characterisation. We have additionally clarified the appropriateness of characterizing a culturable isolate for downstream metabolomic and phenotypic validation on which bioprospecting and biosafety assessment depend. (Revised paragraph 2 of the Introduction.)
2. Reviewer comment: *The manuscript links plant-associated bacteria with antimicrobial resistance dissemination through horizontal gene transfer. However, this argument would benefit from additional explanation regarding: ecological pathways connecting plant microbiomes and clinical settings, evidence supporting gene exchange, relevance of Kluyvera as an environmental reservoir of clinically important resistance genes.* *Otherwise, the transition appears somewhat abrupt.*
**Response: **We agree that this transition was underdeveloped and have substantially revised it. Rather than invoking a general connection between plant microbiomes and clinical settings, we now anchor the argument in the specific, mechanistically documented example most relevant to this organism, that is the CTX-M family of extended-spectrum β-lactamases. We now explain that the chromosomal *bla*KLU genes of environmental, plant-associated *Kluyvera* spp. are the recognised progenitors of the CTX-M family, which has been mobilised onto plasmids by the insertion sequences ISEcp1 and ISCR1 and subsequently disseminated among clinical pathogens (Humeniuk et al., 2002; Cantón et al., 2012; Rodriguez & Gutkind, 2024). We further note that, in *K. ascorbata* specifically, the chromosomal *bla*KLUA gene corresponds to the plasmid-borne CTX-M-2 cluster (Rodriguez & Gutkind, 2024; Yu et al., 2023), which directly frames the CTX-M-2 determinant we report in this genome. This provides the concrete ecological pathway, the evidence of horizontal mobilisation, and the reservoir role of *Kluyvera* that the reviewer requested. We have been careful to state that the documented pathway concerns environmental *Kluyvera*, and that characterising endophytic representatives extends surveillance of this reservoir into the plant-internal compartment rather than implying that endophyte-to-clinic transfer has itself been demonstrated.  (Revised paragraph 3 of the Introduction.)
3. Reviewer comment: *The introduction states that "no complete genome characterisation of an endophytic Kluyvera ascorbata from Piper crocatum has been reported", but this claim should be supported by a more comprehensive literature context. The authors should clearly distinguish their work from previous genome reports of K. ascorbata isolated from environmental, plant-associated, or clinical sources, explaining precisely what knowledge gap this study fills.*
**Response: **We have qualified this statement and now distinguish our isolate from existing K. ascorbata genomes. Publicly available genomes derive largely from clinical or environmental sources (e.g. the type strain ATCC 33433 and reference strain SK, NZ_CP096201.1), whereas no genome of an endophytic *K. ascorbata* from a tropical medicinal plant such as *Piper crocatum* has been reported; this defines the specific gap our resource fills. (Revised final paragraph of the Introduction.)
4. Reviewer comment: *The manuscript should explain why this genome will be valuable beyond the current study—for example, comparative genomics, evolutionary studies, genome mining, natural product discovery, or future surveillance of environmental antimicrobial resistance.*
**Response: **We now state that the genome supports comparative and evolutionary genomics of the *Kluyvera*/CTX-M lineage, genome mining and natural-product discovery from medicinal-plant endophytes, and environmental AMR surveillance. (Added to the Introduction aims and echoed in the Conclusion.)
5. Reviewer comment: *The manuscript does not explain why enrichment and bacterial isolation were performed at 37°C, which is considerably higher than the natural environmental temperature of Piper crocatum. This incubation temperature may preferentially recover mesophilic bacteria with potential clinical relevance while excluding other endophytic taxa. The authors should justify this choice or discuss its implications for isolate recovery.*
**Response: **We agree this warrants comment. 37 °C was chosen to target culturable, fast-growing endophytes compatible with downstream high-molecular-weight DNA extraction and long-read sequencing, and reflects a deliberate focus on mesophilic, potentially clinically relevant taxa. We now acknowledge explicitly that this temperature biases recovery toward mesophiles and away from other endophytic taxa and frame the isolate as one representative of the culturable community rather than a survey of total endophytic diversity. (Added to Methods and noted as a limitation.)
6. Reviewer comment: *Although plating of the final rinse water is reported, the methodology would be stronger if additional evidence of successful surface sterilization were included (e.g., imprint method or molecular verification). The authors should discuss whether plating alone is sufficient to exclude epiphytic contamination*.
**Response: **We thank the reviewer for this point. Surface sterilization was validated by plating an aliquot of the final rinse water, with no growth observed, as one of the standards, widely used culture-based controls in endophyte isolation. We agree that tissue imprinting or amplicon-based verification would provide more stringent confirmation of endophytic origin. However, as the tissue was fully processed during isolation, these controls cannot be applied retrospectively. We have retained an explicit statement of the rinse-plating control in the Methods, and we will adopt imprinting as an additional control in future isolations.
7. Reviewer comment: *The authors describe DNA integrity assessment but do not report the acceptance criteria used before sequencing.*
*Please specify: minimum DNA concentration, A260/A280 ratio, A260/A230 ratio, DNA quantity loaded for library preparation. These parameters improve reproducibility.*
**Response: **We have specified the DNA acceptance criteria applied prior to library preparation, based on the manufacturer's recommended input requirements for the SQK-NBD114.24 kit (minimum concentration of 15 ng/μl, A260/280 of 1.8, A260/230 of 2.0–2.2, and DNA amount of 400 ng). (Revised Methods, “DNA extraction and sequencing.”)
8. Reviewer comment: *The sequencing workflow is comprehensive, but no information is provided regarding: total number of reads, total bases generated, mean read length, N50 read length, sequencing depth (coverage). These values are essential because they directly influence assembly quality.*
**Response: **Most of these are already reported. The first paragraph of Results and Table 1 give 117,809 reads, 174.6 Mbp total, read N50 of 3,007 bp, and ~35× estimated coverage. We have added the mean read length of 1482 for completeness.
9. Reviewer comment: *The manuscript states: "assemblies with >95% completeness and <5% contamination were considered high-quality." However, BUSCO evaluates completeness and duplication—not contamination. Contamination is usually estimated using tools such as: CheckM, CheckM2, GUNC, BlobToolKit. The authors should either replace "contamination" with "duplication," or include an independent contamination assessment. This is one of the most important methodological issues.*
**Response: **We thank the reviewer for catching this; the reviewer is correct that BUSCO reports completeness and duplication, not contamination. We have corrected error and now report an independent contamination estimate. Contamination was assessed with CheckM2 v1.1.0, yielding 0 % contamination and 100 % completeness, confirming a clean assembly. (Added to Methods and Table 1.)
10. Reviewer comment: *The manuscript states: "Results were interpreted based on the established ANI threshold (>90%)." This threshold is incorrect for bacterial species delineation. Current consensus is: 95–96% ANI for species boundaries. The manuscript should revise this statement and cite current ANI standards.*
**Response: **We thank the reviewer for this correction. We have revised the threshold to the current consensus of 95–96% ANI for species boundaries. Our value (98.6% against NZ_CP096201.1) lies well above this boundary, so the species assignment is unchanged. (Revised Methods, "Taxonomic and phylogenomic analysis.")
11. Reviewer comment: *Only Medaka polishing was performed. Please clarify: whether multiple polishing rounds were conducted, whether polishing converged, why no additional polishing (e.g., Racon) was performed.*
**Response: **We thank the reviewer for this comment. We have now clarified that a single round of Medaka was performed. Reads were base called with the Dorado super-accuracy model. With super-accuracy ONT reads, Medaka is the developer-recommended consensus step and Racon is no longer required for this workflow. (Revised Methods, “Sequence processing and assembly.”)
12. Reviewer comment: *Although the Bakta version is reported, the manuscript should indicate: annotation mode, genetic code used, whether pseudogenes were predicted, whether default parameters were employed.*
**Response: **We have added the requested annotation details to the Methods. Genome annotation was performed with Bakta v1.8.2 using the default annotation workflow and the light database (db-light). Specifically: (i) annotation was run in the standard (non-metagenome) mode; (ii) coding sequences were predicted using translation table 11; (iii) pseudogene detection and annotation were enabled by default (the --skip-pseudo-option was not used), so pseudogenes were predicted; and (iv) default parameters were used throughout. (Revised Methods, "Functional annotation and genome mining.")
13. Reviewer comment: *The reported sequencing depth (~35×) is relatively modest for generating a high-quality Oxford Nanopore-only bacterial genome assembly. Although the BUSCO completeness is excellent (100%), the authors should discuss whether this sequencing depth may have contributed to the fragmented assembly (eight contigs) and whether additional sequencing could improve contiguity.* *The authors attribute fragmentation entirely to rRNA operons because the read N50 (~3 kb) is shorter than the operon length. However, this explanation remains speculative. Other factors may also contribute, including repetitive insertion sequences, prophages, tandem repeats, GC-rich regions. The authors should either provide assembly graph evidence supporting this explanation or moderate the wording.*
**Response: **We have moderated the interpretation of the assembly fragmentation and now note that, while the rRNA operons are the principal likely cause, other repetitive elements (insertion sequences, prophages, tandem repeats) may also contribute. We have additionally addressed the sequencing-depth point: we now state that contiguity was limited primarily by read length rather than depth, that the ~35× coverage — though modest — was sufficient to recover the complete gene space (100% BUSCO completeness, 0% CheckM2 contamination), and that a more contiguous assembly would be achieved most effectively by increasing read length rather than depth alone (Added to first paragraph of Results.)
14. Reviewer comment: *The authors attribute fragmentation entirely to rRNA operons because the read N50 (~3 kb) is shorter than the operon length. However, this explanation remains speculative. Other factors may also contribute, including repetitive insertion sequences, prophages, tandem repeats, GC-rich regions. The authors should either provide assembly graph evidence supporting this explanation or moderate the wording.*
**Response: **We have moderated the wording and now present rRNA-operon repeats as the most likely principal contributor while acknowledging that additional repetitive elements (insertion sequences, prophages, tandem repeats) may also limit contiguity. (Revised first paragraph of Results.)
15. Reviewer comment: *The manuscript reports assembly statistics but provides little context. It would strengthen the Results if the assembly quality (genome size, GC content, contig number, N50) were briefly compared with published Kluyvera ascorbata genomes to demonstrate consistency.*
**Response: **We have added a brief comparison confirming that our genome size (4.98 Mbp) and GC content (54.26%) are consistent with published *K. ascorbata* genomes (5.1 Mbp with GC content of 54% for NCBI RefSeq NZ_CP096201.1, and 4.9 Mbp with GC content of 54.5% for ATCC 33433), supporting assembly integrity. (Added to Results.)
16. Reviewer comment: *The statement "Low similarity may reflect either true biosynthetic novelty..." is speculative. Low similarity in antiSMASH can also result from fragmented clusters, incomplete assemblies, annotation differences, limited database representation. The manuscript should avoid implying novelty without experimental validation.*
  We agree and have reinforced the existing caveat. The revised text states that low antiSMASH similarity may equally arise from cluster fragmentation across contigs, annotation differences, under-representation of *Kluyvera*-associated clusters in current reference databases, or biosynthetic novelty, requiring further metabolomic validation. (Revised Results, "Biosynthetic gene clusters.")
17. Reviewer comment: *The manuscript identifies 26 AMR determinants, yet most are regulatory or efflux-associated genes. The wording could lead readers to interpret these as confirmed resistance genes. The authors should emphasize that many identified loci are intrinsic housekeeping genes, regulatory genes, putative contributors to resistance, rather than acquired resistance determinants.*
**Response: **We share this concern and had framed the Strict hits as "predominantly intrinsic *Enterobacteriaceae* efflux systems and chromosomal target-modification genes whose contribution to clinical resistance phenotype cannot be inferred from sequence alone." We have strengthened this to state explicitly that most loci are intrinsic, regulatory, or putative contributors rather than acquired resistance determinants. (Revised Results, "Antimicrobial resistance genes.")
18. Reviewer comment: *The manuscript states "Perfect hit was blaCTX-M-2..." Since Kluyvera species possess chromosomal blaKLU genes that are ancestral to CTX-M enzymes, the authors should clarify whether CARD annotated this locus directly as CTX-M-2, or whether it represents a homologous ancestral chromosomal β-lactamase.*
*This distinction has evolutionary importance.*
**Response: **We have now noted that CARD-RGI returned a Perfect hit (100% identity, full-length) to the CTX-M-2 model. Consistent with *K. ascorbata* being the recognised chromosomal progenitor of the CTX-M-2 sublineage, we note that this chromosomally located locus is best interpreted in the context of the ancestral blaKLUA lineage rather than as an acquired plasmid-borne ESBL. (Revised Results.)
19. Reviewer comment: *Phenotypic antimicrobial susceptibility testing is mentioned only near the end. Since the Results emphasize antimicrobial resistance genes, the absence of MIC or susceptibility testing should be acknowledged immediately after presenting the CARD results.*
**Response: **We have noted the phenotypic-testing limitation directly following the CARD results, stating that MIC/susceptibility testing was outside the scope of this Genome Note and that predicted determinants are not phenotypically confirmed. (Results, "Antimicrobial resistance genes.")
20. Reviewer comment: *The absence of CRISPR/Cas systems is reported without interpretation. A brief statement regarding whether CRISPR absence is common in Kluyvera species would improve biological context.*
**Response: **We have added the statement that CRISPR/Cas-negative result is consistent with previous report of low distribution of CRISPR/Cas systems in the Kluyvera genus, to provide biological context.
21. Reviewer comment: *The final paragraph appropriately states that detailed interpretation is beyond the scope of the Genome Note. However, several earlier paragraphs already provide biological interpretation (e.g., novelty of BGCs, AMR implications). The manuscript should maintain a consistent balance between descriptive reporting and interpretation.*
**Response: **We have harmonised the tone: interpretive statements (BGC novelty, AMR implications) are now consistently hedged and framed as prediction requiring validation, so that they align with the closing statement that detailed functional interpretation is beyond the scope of a Genome Note. (Revised throughout Results and Conclusion.)
22. Reviewer comment: *The conclusion should be objective, with further perspective, or should add at least a few sentences about future study/future perspective.*
**Response: **We have revised the Conclusion to state findings objectively and added a forward-looking paragraph outlining future work: metabolomic validation of the predicted BGCs, phenotypic AST to confirm the predicted resistome, and comparative genomics across endophytic and clinical Kluyvera to trace CTX-M evolution. (Revised Conclusion.)
23. Suggested references
**Response: **We have expanded the relevant literature throughout the manuscript where it strengthens the argument.

We are grateful for the reviewer's expertise and thorough evaluation, which has improved the rigour and clarity of the manuscript. We hope Version 2 addresses all the reservations raised.
